# Current State and Future Trends: A Citation Network Analysis of the Orthokeratology Field

**DOI:** 10.1155/2019/6964043

**Published:** 2019-03-07

**Authors:** Miguel Angel Sanchez-Tena, Cristina Alvarez-Peregrina, Jose Sanchez-Valverde, Cesar Villa-Collar

**Affiliations:** ^1^Faculty of Biomedical and Health Sciences, Universidad Europea de Madrid, Madrid 20822, Spain; ^2^Clinica Oftalmologica Doctor Lens, Madrid 28009, Spain

## Abstract

**Introduction:**

Citation network analysis is a powerful tool that allows for a visual and objective representation of the past, present, and potential future directions of a research field. The objective of this study is using citation analysis network to analyse the evolution of knowledge in the field of orthokeratology.

**Materials and Methods:**

The database used in this citation networks analysis study was Scopus. The descriptor used was “orthokeratology” limited to three fields: title, keywords, and/or abstract, analysing the five most cited authors. Only articles cited at least twenty times were used. The computer software used was UCINET with two types of analysis, qualitative and quantitative.

**Results:**

27 nodes have been included according to the search and inclusion criteria. In qualitative analysis, based on illustrate results, the relationships among nodes and their positions and connections show how the study of Cho et al. in 2005 is clearly positioned as a central cutoff point in the network. Quantitative analysis reveals the normalized value of the sample and shows how the study of Cho et al. in 2005 presents the highest percentage of input connections.

**Conclusions:**

This study shows the state of the flow of information in the orthokeratology field by providing links in bibliographic citations from a qualitative and quantitative point of view.

## 1. Introduction

Orthokeratology is a nonsurgical technique that has evolved in the last two decades, starting as a limited clinical treatment, to become a real alternative to reduce, modify, or eliminate refractive errors.

Nowadays, there is enough evidence on the capability of orthokeratology to slow down myopia and the elongation of the eyeball in children [1]. It is therefore an important topic in current scientific research as evidenced by a recent published bibliometric study [2]. This study highlights the boom in orthokeratology research activity over the past 5 years, as well as the growing interest among the scientific community. However, bibliometric studies have some limitations, and they need to be completed with other kinds of analyses of scientific production as citation networks' analysis.

Citation network analysis is a powerful tool that gives a visual and objective representation of the past, present, and potential future directions of a research field [3]. This kind of analysis gives essential information to identify knowledge gaps, trends, and relationships among scientific research in any topic, being helpful in moving any field forward.

The objective of this study is using citation analysis network to analyse the evolution of knowledge in the field of orthokeratology.

## 2. Materials and Methods

The database used in this citation networks analysis study was Scopus. The descriptor used was “orthokeratology” limited to three fields: title, keywords, and/or abstract, analysing the five most cited authors. Only articles cited at least twenty times in Scopus were selected.

According to the above criteria, a list was obtained, in which each positive article became a node of the citation network. The number in the list depended on how many times the article had been cited, the most cited articles being on the top of the lists. These data were collected on Excel, including all the references of each article and remarking which of the other positive articles of the list are cited, through the link “who quotes whom.”

Using the computer software UCINET, the binary and asymmetric square matrix was defined, being 0 = no cited and 1 = cited. The attributes were defined by author, year of publication, and title. Each one was classified within a category: control of myopia or safety and efficacy.

Once the elaborated matrix was available, qualitative and quantitative analysis started.

Qualitative analysis started with the elaboration of a network graph with a UCINET NetDraw program assistant. The structure of the network is analysed from four grouping measures: clique, N-clique, N-clan, and K-plex.

Clique [4–6] is a subgroup within the graph formed by a set of nodes that have all the possible links between them; usually, each clique is formed by three or more members. For N-clique [4–6], a node is a member of a clique if it is connected to all its members at a distance greater than one. The value two is used in all members of the subgraph that do not need to be adjacent but are reachable by an intermediary. To restrict connectivity through nodes that are not members of the clique, N-clan [4–6] is used in which the relationship is still measured, that is, someone is cited through another, but these citations must be reached by other members of the clique.

K-plex [4–6] analysis shows different visions of substructures of the network since it highlights social circles superimposed. It is a subgraph in which each node is adjacent to all but a certain *K* number of other nodes. A node has a *K* value of two if it cites all its members except two of them, eliminating the presence of intermediaries. The grouping measures are complementary, and the combination of them shows a better idea of the structure of the network.

Once grouping analysis was carried out, the breakpoints of the structure of the relationships established are analysed by quantifying the subgroups that are not connected to the rest.

For the quantitative analysis, links are analysed through three measures of centrality, giving the position of the nodes within the network and the structure of the network under study: range, degree of intermediation, and degree of proximity.

Range [4–6] (DEGREE) is the number of direct links that a node has. There is an entry range (times the node receives a link from another) and exit range (times the node sends a link to another). As it is an oriented asymmetric matrix formed by digraphs or pairs of nodes, it quantifies which node is most strongly connected.

The Nrmdegree value indicates the normalized range or percentage of connections that a node has over the total network.

The betweenness degree [4–6] (BETWEENNESS) shows when a node connects other two. The interaction between nonadjacent nodes may depend on others, which implies that they can exercise some control over them. This is important for the analysis of the centrality and the cohesion of the network. If we eliminate a node, we can see clearly whether it is a cutoff point or not, measuring how many times a node interposes among others in its geodetic distance.

The closeness degree [4–6] (CLOSENESS) is the distance of a node with the rest, measuring the geodetic distance of all the nodes. In this way, the closer a node from the others is, the greater the index of centrality will be and the faster it will be able to access the information.

## 3. Results

All searches were carried out in September 2018.

Table 1 shows the 27 nodes that have been included according to the search and inclusion criteria. They have been classified according to their attributes within one of the established categories.

**Figure 1:**
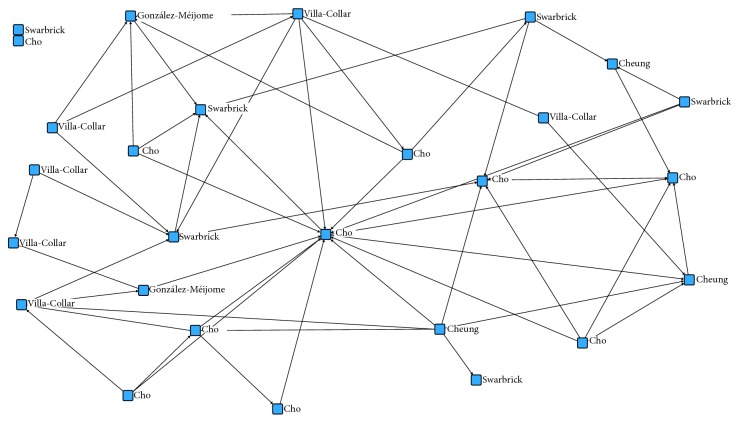
Network graph.

Figure 1 illustrates results from qualitative analysis, explaining the relationships among nodes and their positions and connections. It shows that Cho et al. [7] is clearly positioned as a central cutoff point in the network. This makes a vulnerable network but not disconnected, as there are links between peripheral nodes.

Another key point in Figure 1 is that there are groups and subgroups within the net, making “subgraphs” with more than three linked nodes. UCINET software, through the relational matrix, found 14 cliques but only one increases to four members. The union of Cho et al. [7] and Chen et al. [16] is the most important subgroup in the network, as these authors share papers and relevance in the orthokeratology field.

To N-clique, there are only two disconnected nodes, Swarbrick [10] and Choy et al. [29]. There are 17 related groups, eight of them having more than nine members. N-clan finds 12 groups, four of them made up of nine members, so N-clan has very similar results to N-clique.

In the K-plex analysis, 139 subgroups were calculated, 29 of them having more than three members. This result provides little information beyond the strong cohesion of the citations in this field and the large number of subgroups overlapping within the network. The presence of Cho [7] is observed in all the subgroups found.

Quantitative analysis reveals the normalized value of the sample Nmdegree (DEGREE). It shows that the study of Cho et al. [7] presents the highest percentage of input connections. Also, the study of Chen et al. [16] is the one with the highest percentage of exit degree, being the one that includes the most articles in its references belonging to the network.

The highest value of betweenness (BETWEENNESS) in our network is the study of Cho et al.[7]. It unites the network and is the cutoff point, structuring the network around it.

The degree of closeness (CLOSENESS) presents very close values among all the components of the network, despite the study of Cho et al. [7] being the most valuable as all values of centrality show still.

## 4. Discussion

Through qualitative analysis, it is proved that the net is not disconnected, although there is a very strong cutoff point in Cho et al. [7]. Just looking to the graph, we can observe that the net is not very dense and that the paper of Cho et al. [7] is the most strongly connected to others and has the most central position.

Regarding citation structure and cohesion, it is noted that the paper of Cho is the one with more shared cliques in all the measures, being the only cutoff point in the network.

N-clique values show just two papers disconnected, which indicates cohesion across the net. This is because orthokeratology is a very specific field, and all papers tend to mention other articles related to their subject of study. N-clan reinforces the cohesion data of the network, sharing very similar results with N-clique. However, they complement each other, ensuring that these articles maintain a direct relationship.

K-plex continues to highlight the presence of Cho et al. [7] in all the groups what can be understood by the small number of articles included. This makes even clearer the great relevance that this article had in the development of orthokeratology.

The numerous groupings within a very little dense network again indicate a great cohesion. Despite this low density, this kind of analysis reveals the uniformity in the publications related to orthokeratology and the common criteria that authors follow within it. It also suggests the need in future research for the inclusion of broader cohesion measures in these analyses to avoid biases in the data due to lack of density in the network.

Regarding quantitative analysis, degree of centrality corroborates that the study of Cho et al. [7] has the highest degree of entry and exit cites, ensuring that it is the most relevant and influence article in relation to myopia control and orthokeratology. Its betweenness value compared to other published studies in this field, together with the study Chen et al. [16], makes a binomial of relevance for the topic of control of myopia and orthokeratology. Both have common publications and are the network cutoff point, in addition to being the most relevant subgroup.

In relation to the degree of closeness, it shows that the studies of Cho et al. [19] and Swarbrick et al. [8] are the closest to the others. However, they are very far apart, giving two different visions in the orthokeratology field. On the one hand, we found the articles that look for the topic of control of myopia and on the other hand, those that study the effectiveness of the treatment for the correction of refractive errors.

Analysing in detail all the papers, we found eight studies in the category of myopia control. Six of them have been published in the same institution by Cho et al. [7, 9, 15, 16, 18, 19].

In chronological order, these researchers started writing about their daily clinical experience as leaders in the orthoK field. In 2004, they exposed the results of a study about the influence of orthoK on eye axial length [19]. In 2005, they published the most cited study, LORIC [7], that has become a reference in the use of orthoK as a method to control myopia.

In 2012, Villa-Collar leads MCOS Study [11]. MCOS is like the studies previously cited but carried out in Spain instead of Hong Kong. They analysed refractive and biometric changes.

In relation to the category safety and efficacy, we found 19 papers that met the inclusion criteria, Swarbrick et al. standing out with six papers published by each one [8, 10, 13, 14, 17, 22, 24, 26, 28, 29, 31, 33]. They are followed by Villa-Collar with five papers [20, 21, 27, 30, 32].

Swarbrick et al. is a pioneer with her study in 1998 [8] about corneal response to orthoqueratology. This is in concordance with the data about the degree of closeness.

Later studies of Swarbrick focussed on the incidence of microbial keratitis, with a review and an update about orthoqueratology in 2006 [10].

Villa-Collar focused on studying short- and long-term effects of orthoK on corneal cells morphology, on the effects on tear film, and on quality of life of orthoK wearers versus other methods.

All the papers exposed in this citation network research give a valuable and relevant data that justify that these papers have been the most cited. Most of the researchers are pioneers in publishing papers about different uses of orthoK and about changes in eye and vision produced by orthoqueratology.

## 5. Conclusions

In conclusion, this study highlights the state of the flow of information in the orthokeratology field by providing links in bibliographic citations from a qualitative and quantitative point of view.

## Figures and Tables

**Table 1:** Attributes network.

Node	Authors	Title	Year	Categories
1	Cho et al. [7]	The longitudinal orthokeratology research in children (LORIC) in Hong Kong: A pilot study on refractive changes and myopic control	2005	Myopia control
2	Swarbrick et al. [8]	Corneal response to orthokeratology	1998	Safety and efficacy
3	Cho and Cheung [9]	Retardation of myopia in orthokeratology (ROMIO) study: A 2-year randomized clinical trial	2012	Myopia control
4	Swarbrick [10]	Orthokeratology review and update	2006	Safety and efficacy
5	Santodomingo-Rubido et al [11]	Myopia control with orthokeratology contact lenses in Spain: refractive and biometric changes	2012	Myopia control
6	Queirós et al [12]	Peripheral refraction in myopic patients after orthokeratology	2010	Myopia control
7	Watt and Swarbrick [13]	Microbial keratitis in overnight orthokeratology: review of the first 50 cases	2005	Safety and efficacy
8	Watt and Swarbrick [14]	Trends in microbial keratitis associated with orthokeratology	2007	Safety and efficacy
9	Charm and Cho [15]	High myopia-partial reduction orthok: a 2-year randomized study	2013	Myopia control
10	Chen et al. [16]	Myopia control using toric orthokeratology (to-see study)	2013	Myopia control
11	Boost and Cho [17]	Microbial flora of tears of orthokeratology patients, and microbial contamination of contact lenses and contact lens accessories	2005	Safety and efficacy
12	Cheung et al. [18]	Asymmetrical increase in axial length in the two eyes of a monocular orthokeratology patient	2004	Myopia control
13	Cho et al. [19]	Practice of orthokeratology by a group of contact lens practitioners in Hong Kong: Part I. General overview	2002	Myopia control
14	Nieto-Bona et al. [20]	Short-term effects of overnight orthokeratology on corneal cell morphology and corneal thickness	2011	Safety and efficacy
15	Nieto-Bona et al. [21]	Long-term changes in corneal morphology induced by overnight orthokeratology	2011	Safety and efficacy
16	Chen et al. [22]	A pilot study on the corneal biomechanical changes in short-term orthokeratology	2009	Safety and efficacy
17	Cheung and Cho [23]	Subjective and objective assessments of the effect of orthokeratology-a cross-sectional study	2004	Safety and efficacy
18	Cho et al. [24]	An assessment of consecutively presenting orthokeratology patients in a Hong Kong based private practice	2003	Safety and efficacy
19	González-Méijome et al. [25]	Pilot study on the influence of corneal biomechanical properties over the short term in response to corneal refractive therapy for myopia	2008	Safety and efficacy
20	Jayakumar and Swarbrick [26]	The effect of age on short-term orthokeratology	2005	Safety and efficacy
21	González-Pérez et al. [27]	Tear film inflammatory mediators during continuous wear of contact lenses and corneal refractive therapy	2012	Safety and efficacy
22	Lum et al. [28]	Mapping the corneal sub-basal nerve plexus in orthokeratology lens wear using in vivo laser scanning confocal microscopy.	2012	Safety and efficacy
23	Choy et al. [29]	Effect of one overnight wear of orthokeratology lenses on tear composition	2004	Safety and efficacy
24	Queirós et al. [30]	Effect of pupil size on corneal aberrations before and after standard laser in situ keratomileusis, custom laser in situ keratomileusis, and corneal refractive therapy	2010	Safety and efficacy
25	Cho et al. [31]	Non-compliance and microbial contamination in orthokeratology	2009	Safety and efficacy
26	Queirós et al. [32]	Quality of life of myopic subjects with different methods of visual correction using the NEI RQL-42 questionnaire	2012	Safety and efficacy
27	Chen et al. [33]	Posterior corneal curvature changes and recovery after 6 months of overnight orthokeratology treatment	2010	Safety and efficacy

## Data Availability

The data used to support the findings of this study are available from the corresponding author upon request.
